# Effects of physical exercise in sarcopenia on patients undergoing bariatric surgery: A protocol for a randomized clinical trial

**DOI:** 10.1016/j.mex.2023.102043

**Published:** 2023-01-28

**Authors:** Cláudia Amaro Santos, Ana Margarida Cinza, Ânia Laranjeira, Margarida Amaro, Manuel Carvalho, Jorge Bravo, Sandra Martins, Armando Raimundo

**Affiliations:** a Hospital Espírito Santo de Évora, EPE, Évora, Portugal; b CRI.COM – Centro Responsabilidade Integrada de Cirurgia da Obesidade e Metabólica, Évora, Portugal; c CHRC - Comprehensive Health Research Centre, Universidade de Évora, Évora, Portugal; d Departamento de Desporto e Saúde, Escola de Saúde e Desenvolvimento Humano, Universidade de Évora, Portugal; e Universidade Europeia, Lisboa, Portugal; f Instituto de Saúde Ambiental (ISAMB), Faculdade de Medicina da Universidade de Lisboa, Lisboa, Portugal

**Keywords:** Exercise, Bariatric surgery, Fat-free mass, Sarcopenia, Skeletal muscle mass, EXPOBAR

## Abstract

Severe obesity is a chronic disease and bariatric surgery is the treatment with more proven efficacy in reducing weight. After surgery, the weight loss is greatly associated with a significant reduction of skeletal muscle and bone mineral mass, with an increased risk of sarcopenia for these patients. Prophylactic programs that prevent sarcopenia in bariatric surgery patients seems to be one of the crucial points for the long-term surgical success of bariatric and metabolic surgery. This article aims to describe a protocol using supervised exercise applied after bariatric surgery on skeletal muscle mass index, body composition and strength to determinate sarcopenia in bariatric patients. A RCT will be conducted with 46 patients. Baseline measures will be compared with measures after de exercise program, in five different chronologic times. Participants will be randomly allocated to: 1) combined exercise group or 2) control group. The intervention will be 16 weeks for a combined exercise, started 1 month after surgery. The present study is expected to generate significant information about the role of exercise in patients undergoing bariatric surgery.

## Graphical Abstract

**Figure:**
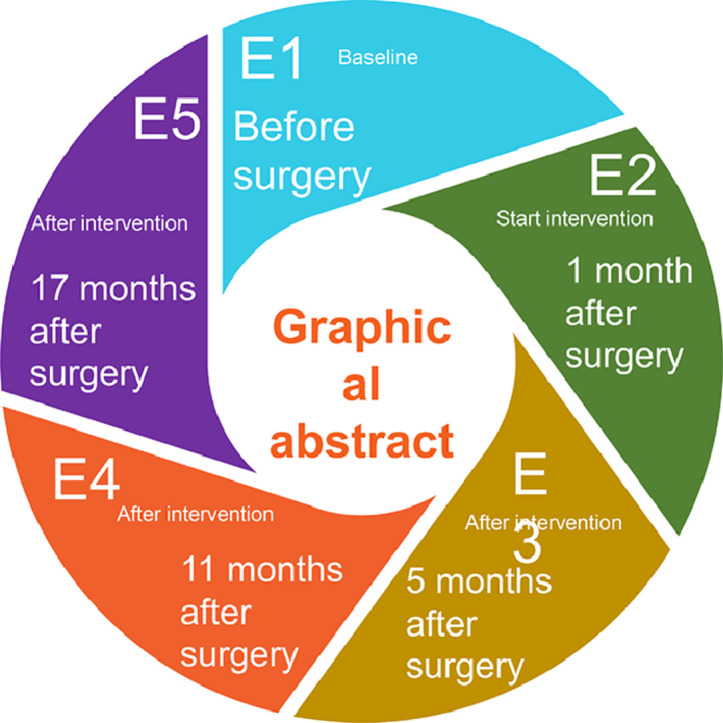

**In separated document**

Specifications table

**Table:** 

Subject area	Medicine and Dentistry

More specific subject area	Assessment of skeletal muscle mass index, body composition and strength to determinate sarcopenia in bariatric patients
Name of your protocol	EXPOBAR
Reagents/tools	DEXA (DXA, Hologic QDR, Hologic, Inc., Bedford, MA, USA) (Biodex®, System 3 Pro, Biodex Corp., Shirley, NY, USA) Handrip Chair 6 minutes’ walk teste circuit
Experimental design	Experimental randomized controlled trial (RCT), open-label, phase III-type study with 45 patients in HD. Protocol using supervised exercise applied after bariatric surgery on skeletal muscle mass index, body composition and strength to determinate sarcopenia in bariatric patients. A RCT will be conducted with 45 patients. Baseline measures will be compared with measures after de exercise program, in five different chronologic times. Participants will be randomly allocated to exercise group or control group. The intervention will be 16 weeks for a combined exercise, started 1 month after surgery.
Trial registration	The study protocol was registered in the Clinicaltrials.gov NCT03497546
Ethics	The study protocol was submitted to the Research Ethics Committee of the University and Hospital and approved under No. 21051
Value of the Protocol	- This protocol is important to evaluate the effects of supervised and structured physical exercise on possible sarcopenia induced by bariatric surgery. - There is a lack of knowledge about the effects of combined exercise on long term after bariatric surgery - This article is important to contribute to the recommendations of the practice of exercise after bariatric surgery.

## Description of protocol

Bariatric surgery is one of the treatments for severe obesity, effective on reducing weight and diseases associated with obesity. After bariatric surgery, weight loss is greatly associated with a significant reduction of skeletal muscle and bone mineral mass, which leads us to induce that after bariatric surgery, patients incur an increased risk of sarcopenia. Prophylactic programs are need for prevent sarcopenia in bariatric surgery patients and seems to be one of the crucial points for the long-term surgical success of bariatric and metabolic surgery. The aim of this randomized clinical trial is to analyze the effects of a 16-week supervised exercise intervention program on the prevention of sarcopenia, after bariatric surgery. As a secondary purpose, it is also intended to characterize metabolic risk factors, physical fitness, and quality of life in post-bariatric surgery patients.

### Study design

A randomized clinical trial, registered as EXPOBAR, will be conducted and the SPIRIT (Standard Protocols Items: Recommendations for Interventional Trials) recommendations were followed, to evaluate the effectiveness of the intervention.

### Subjects

Participants will be selected from the list for surgical intervention in the hospital.

### Inclusion and Exclusion Criteria

As inclusion criteria, patients should be enrolled for bariatric surgery at the hospital, aged between 18 years and 60 years, body mass index between 30 and 50Kg/m2, men and woman, without contraindication to the practice of exercise and agree to participate in the study.

Will exclude patients with problems in locomotion, with previous bariatric surgery, with bariatric surgery complications and, psychiatric diseases or disorders.

### Sample size calculation

The sample size was calculated by the Gpower, assuming an alpha error of 0.05 and a power of 95%, a total of 46 patients will be needed to detect an effect (between group difference) of at least 0,7 standard deviations in the outcome risk of sarcopenia (1). Anticipating a potential 20% lost to follow-up and based on the number of follows in our center, a total of 55 patients will be recruited and will be randomized into Control Group (CG) and Intervention Group (IG). Exercise training will begin one month after surgery, with a three times per week frequency, up to a maximum of 55 minutes per session.

### Recruitment

The invitation to participate will be made in the context of consultation and participants who agree to participate in the study will be delivered the free and informed consent form, previously approved by the University and Hospital Ethics Committee.

### Randomization

Each participant will be randomly assigned to each group after signing the informed consent and conducting the initial assessments. All laboratory samples and data collected will be identified with identification ID, safeguarding the confidentiality of the collected data.

At the end of this study, all participants of the control group will be offered the same intervention as the exercise group.

### Procedures

**Fig. 1:**
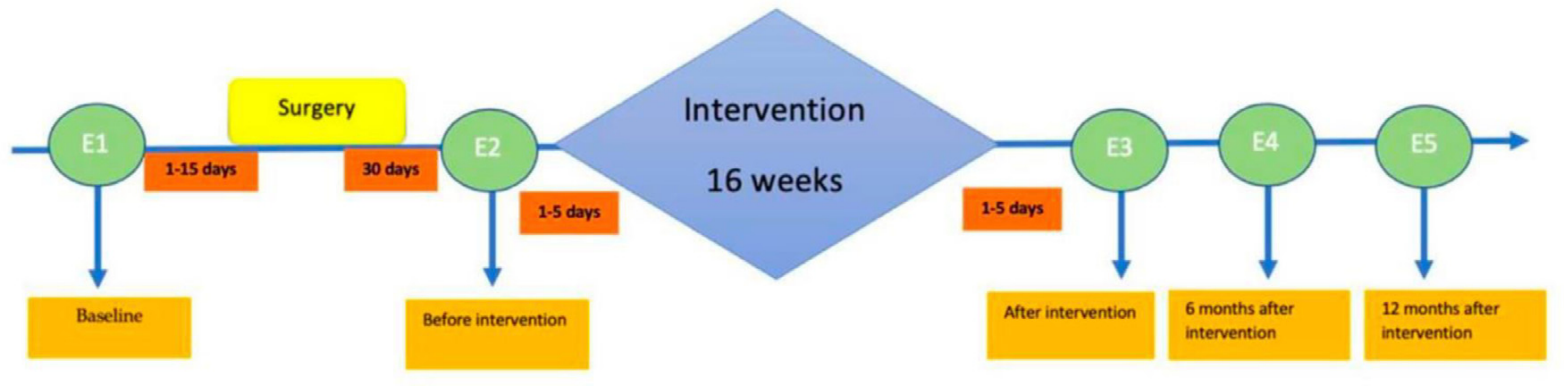
Training schedule.

Immediately after recruitment, all enrolled patients who agree to participate in the study will sign the informed consent form. The subjects will then proceed to the baseline data collection, intervention protocol and final assessments (Fig. 1).

Weight evaluation will be done using a scale and height of a stadiometer. Based on these values, the body mass index will be calculated, and the abdominal circumference will be determined by a measuring tape.

Metabolic risk factors will be determined by clinical analyses (blood sample) performed in the context of routine surgical evaluation, with the determination of inflammatory markers. The mean blood pressure will be evaluated by a digital sphygmomanometer. Through the analytical profile will be determined the hormonal profile, since leptin and ghrelin concentrations

Saliva collection will be made at the moments of evaluation, for a small recipient during five minutes, which will be analyzed by the biochemistry department.

The glycemia variation will be done through an implantable 24-hour monitoring device for 5 days.

To evaluate body composition, the Dual-energy X-ray absorptiometry - DEXA (DXA, Hologic QDR, Hologic, Inc., Bedford, MA, USA) device will be used to measure the % fat mass, muscle mass and bone mass [1].

The muscle strength of the upper limbs will be evaluated by manual pressure dynamometry (Handgrip) in both hands, with a maximum contraction of five seconds. The muscle strength of the lower limbs will be evaluated by the sit to stand test, in which participants will be instructed to stand and sit for 30 seconds, as many times as possible. The strength of lower limbs, as well as muscle fatigue, will be evaluated with an isokinetic dynamometer (Biodex®, System 3 Pro, Biodex Corp., Shirley, NY, USA) using a protocol with two series, the first of which is 6 repetitions at 60°/sec. and the second with 25 repetitions at 180°/sec [2].

Cardiorespiratory fitness will be assessed using the 6-minute walk test (TC6) [2].

Sedentary Behavior and Physical Activity will be measured by accelerometer, through the feature of the application of accelerometers (ActiGraph GT3X model, Fort Walton Beach, Florida, USA) for 5 days before the surgery and three times after the exercise program [3].

Questionnaire "Bariatric Analysis and Reporting Outcome System (BAROS) as a life quality self-report measure, validated for Portuguese, specific for bariatric surgery.

### Intervention description

The exercise program will cover a combination of aerobic and strength training, based on other experimental studies already developed with morbidly obese patients, but also following the Consensus on Exercise Reporting Template (CERT) [4].

The duration of the program is 16-weeks, 3-times a week, for up to 50 minutes per session, starting 1 month after surgery, based on the recommendations of the WHO and the ACSM, because the guidelines for morbidly obese patients undergoing bariatric surgery are not defined. Information on exercises for morbidly obese adults is limited, so the exercise programs will follow the guidelines for adults aged 18 to 65 years healthy, with chronic diseases or disabilities [5].

In recent recommendations, those who have chronic diseases, or some type of disability should start by doing small amounts of physical activity with a gradual increase in frequency, intensity, and duration. In addition, for additional benefits should do strengthening activity involving all major muscle groups and moderate or high intensities, at least 2 days a week. As general recommendations, a combination of intensities throughout the week, 150 to 300 minutes of moderate-intensity physical activity or 75 to 150 minutes of vigorous-intensity physical activity [6].

High-intensity interval training programs typically involve short periods of high-intensity exercise followed by a short period of rest or active recovery. Interval training is a type of training, which consists of alternating between periods of moderate to high-intensity exertion and rest, with variable duration, according to the exercise performed and the objective of the person. This type of training has been shown to be more beneficial to improve abdominal fat and body weight while maintaining muscle mass., in increasing weight loss, as well as a positive effect on bone mineral density, aerobic capacity and muscle strength [7].

Exercise prescription includes the type, intensity, duration, frequency, and progression of physical activity. These five components are applicable to the development of exercise programs for persons regardless of age, functional capacity, and presence or absence of coronary heart disease risk factors. These five components of exercise prescription are reported as Frequency, Intensity, Time, and Type (FITT) with the Volume of exercise added along with the Progression component to produce the acronym FITT-VP. The training sessions (Table 2) will follow an evolution subdivided by progressive phases in training (Table 1). As carried out in previous studies, this strategy carried out through phases of increment of training variables allows better adaptability for this type of patients [8, 9, 10].

**Table 1:** Training protocol.

Week 1	Week 2	Week 3	Week 4	Week 5	Week 6	Week 7	Week 8	Week 9	Week 10	Week 11	Week 12	Week 13	Week 14	Week 15	Week 16
Phase 1 – Training resistance				Phase 2 – Training Hypertrophy						Phase 3 – Training Strength					

Each session will start with 5 minutes of warm-up and finalization with 10 minutes of a cool-down, with work of flexibility and proprioception. The maintenance of balance and postural stability may be compromised in obese individuals, depending on the degree of obesity, although the support base provided by the position of the foot is proportional to the structural morphology of each subject. Flexibility is also gradually impaired in obese individuals and of course, these changes may be related to postural changes aggravated by a sedentary lifestyle and biological aging itself alongside all metabolic alterations inherent to the pathology of obesity [11].

And the warm-up and the cool-down will be developed as the component of training with the evolution by phases, both in time and in intensity. The first phase will include 20 minutes of interval training, encompassing circuit strength training. Each phase will have an increment of 10 minutes in the central block, always with a prior evaluation of the patient's response.

### Evaluation

We have five evaluations, baseline (before surgery), before the program (1 month after surgery), after the program (5 months after surgery), 6 months after the program (11 months after surgery) and 12 months after the program (17 months after surgery), as show in Table 2. De CG will be evaluated at the same time that the IG (Fig. 1).

**Table 2:** EXPOBAR protocol.

	Intervention group	Control group
1st Evaluation Before Surgery	Baseline	Baseline
2nd Evaluation Before the Program	1 month (post-surgery)	1 month (post-surgery)
3rd Evaluation After the Program	5 months (post-surgery)	5 months (post-surgery)
4th Evaluation	11 months (post-surgery) 6 months (post program)	11 months (post-surgery) 6 months (post program)
5th Evaluation	17 months (post-surgery) 12 months (post program)	17 months (post-surgery) 12 months (post program)

## Discussion

EXPOBAR aims to be the first RCT in Portugal to evaluate the effects of supervised and structured physical exercise on possible sarcopenia induced by bariatric surgery. Previous studies suggest that there is a decrease in sarcopenia in the immediate period after bariatric surgery when patients have a record of physical exercise.

Interval training has proven to be the most effective in fat mass loss and in preventing muscle mass loss after bariatric surgery. Also infers an improvement in the cardiometabolic condition, with decreased risk factors.

In addition, we intend to contribute to the recommendations of the practice of exercise after bariatric surgery.

## Statistical methods

Statistical software will be used to determine the parameters to be evaluated. Data normality will be assessed with the Shapiro-Wilk test and will be used an independent t-test or the cui-squared test, to examine differences between groups. To compare dependent variables, a two-way ANOVA will be used considering group (intervention group and control group) and five time points (pre- and post-intervention).

## Ethics statements

The work described has been carried out in accordance with the Ethical Committee of the University and Ethical Professional of the Hospital, approved with ethics protocol number 2105. Participation was voluntary, with written informed consent obtained from all participants.

## CRediT authorship contribution statement

**Cláudia Amaro Santos:** Conceptualization, Methodology, Software, Data curation, Writing – original draft. **Ana Margarida Cinza:** Visualization, Investigation. **Ânia Laranjeira:** Visualization, Investigation. **Margarida Amaro:** Visualization, Investigation. **Manuel Carvalho:** Supervision, Software, Validation. **Jorge Bravo:** Supervision. **Sandra Martins:** Supervision, Software, Validation. **Armando Raimundo:** Supervision, Software, Validation.

## Declaration of interest

The authors declare that they have no known competing financial interests or personal relationships, which have or could be perceived to have influenced the work reported in this article.

## Data Availability

I have shared the link of my data
